# Sustainability innovations: a proposal for an analytical framework and its empirical application in the Schorfheide-Chorin Biosphere Reserve

**DOI:** 10.1007/s11625-022-01241-9

**Published:** 2022-11-17

**Authors:** Caroline Hélène Dabard, Carsten Mann

**Affiliations:** 1 Eberswalde University for Sustainable Development, Schicklerstraße 5, 16225 Eberswalde, Germany; 2 Leuphana University, Lüneburg, Germany

**Keywords:** Innovation, Sustainability transitions, Innovation systems, Social–ecological systems, Rural innovation, Biosphere reserve

## Abstract

Sustainability innovations influence societal transformations through the development of new products, processes, organizations, behaviors or values. Although various research approaches have tackled technological innovations in the last few decades, the specificities and enabling conditions of individual sustainability innovations remain rather unknown. We therefore propose an analytical framework, built on learning from the social–ecological systems and transitions literature. The sustainability innovation framework features four dimensions: context, actors, process and outcomes, which are detailed in 31 variables. We use the sustainability innovation framework to analyze two case studies selected in the Schorfheide-Chorin Biosphere Reserve, Germany. The first refers to technological and organizational innovation in mobility, while the second relates to social and organizational innovation in agriculture. As a result, we highlight commonalities and differences in enabling conditions and variables between the two cases, which underpin the influence of trust, commitment, resource availability, experimenting, learning, advocating, and cooperating for innovation development. The cases further demonstrate that sustainability innovations develop as bundles of interdependent, entangled novelties, due to their disruptive character. Their specificity thereby resides in positive outcomes in terms of social–ecological integrity and equity. This study therefore contributes to transitions studies via a detailed characterization of sustainability innovations and of their outcomes, as well as through a generic synthesis of variables into an analytical framework that is applicable to a large and diverse range of individual sustainability innovations. Further empirical studies should test these findings in other contexts, to pinpoint generic innovation development patterns and to develop a typology of sustainability innovation archetypes.

## Introduction

The buzzwords “eco-innovations,” “green innovations” or “sustainability-oriented innovations” have garnered much attention in research and policy in the last few decades, as they seem to be crucial milestones for societal change (Kratzer 2019; O'Brien and Sygna 2013). Yet, what really is sustainable in the processes at stake and their outcomes currently remains somewhat vague. In recent business-driven understandings of sustainability innovations (Adams et al. 2016; OECD/Eurostat 2018; Varadarajan 2017), there is neither a holistic understanding of sustainability nor much consideration for other types of innovations besides product, process, organizational and marketing innovations (Gamito and Madureira 2019).

In the last few decades, as global changes have placed pressure on the role and potential of innovations for sustainable change, the sustainability transitions research field has addressed innovations as multi-scalar processes within complex systems (Rakas and Hain 2019), thereby adopting a systemic and plural understanding of innovation. A guiding assumption has been that solving environmental issues requires not only changes in technology, but also holistic changes in systems comprised of actors, institutions and technology (Loorbach et al. 2017). A prominent framework in this regard is the multi-level perspective (MLP), which unravels innovation journeys within multi-scalar socio-technical systems (Geels 2002). In addition, the MLP provides an analytical framing for multi-dimensional change processes, by addressing dynamic actor–institution interactions and including issues of agency, normativity, change and stability phases, contextual path dependencies, barriers and windows of opportunity (Geels 2002; Geels and Schot 2007). Another framework, developed in parallel to the MLP, is the technological innovation systems (TIS) approach (Hekkert et al. 2007). Similarly, TIS acknowledges the systemic interactions between actors, networks and institutions in the context of socio-technical systems and focusses on radical cross-sectoral innovations, thereby emphasizing markets, actors and network interactions (Markard and Truffer 2008). With a prescriptive—rather than analytical—purpose, the transition management approach (TM) is a practice-oriented framework that guides innovation processes and navigates transitions in the making (Loorbach 2007; Wittmayer et al. 2018). A novel approach is the geography of sustainability transitions (GeoST) (Coenen et al. 2012), which largely builds on learnings from the MLP and the TIS approaches and also adopts a systemic approach to change. However, rather than focusing on technology, GeoST calls for a space-sensitive analysis of focal innovations, thereby paying close attention to spatial patterns and geographical influencing factors across local and global scales (Binz et al. 2020).

While transitions scholars understand sustainability innovations in terms of socio-technical system changes, an identified blind spot is the meanings and implications attributed to the term *sustainability* (Schlaile et al. 2017). What makes transitions and innovations *sustainable* remains often implicit, and positive impacts are usually taken for granted rather than thoroughly studied (Baker and Mehmood 2015;﻿ Feola 2020; O'Brien and Sygna 2013; Salomaa and Juhola 2020).

Through this article, we contribute to the definition of sustainability innovations and to the conceptualization of an analytical framework for a better understanding of their working conditions. We thereby focus on single innovations—rather than on systemic changes at niche and regime levels, to highlight relevant influences on their development and sustainability outcomes, in a context-specific way. We specifically address the following research questions: (1) What are the characteristics of sustainability innovations? (2) Which factors influence sustainability innovations? and (3) What are the specific outcomes of sustainability innovations? To answer these questions, we unravel the specificities of sustainability innovations and underlying social–ecological and multi-level interactions.

This article is structured as follows. In “Analyzing sustainability innovations”, we define sustainability innovations as multi-scalar and multi-actor processes that develop novel ways to define and meet social needs in a specific context. We then present the theoretical foundations for our analytical framework. The framework features four dimensions of sustainability innovations, namely context, actors, process and outcomes, as well as 31 potential influencing factors and characteristics of sustainability innovations to be assessed in focal case studies. This section builds on learnings not only from the sustainability transitions literature and innovations studies, but also on social–ecological systems research (McGinnis and Ostrom 2014; Ostrom 2007) and sustainability assessments (Gibson 2006). “Methods” displays our case study methods on two sustainability innovations from Schorfheide-Chorin Biosphere Reserve (Germany), to test the analytical framework and gain a better understanding of influences and outcomes. “Results” presents the following results. We discuss our insights and the potential and limitations of our research in “Discussion” and conclude in “Conclusion”.

## Analyzing sustainability innovations

In this section, we first present a capacious definition of sustainability innovations, followed by an analytical framework for the detailed study of focal sustainability innovations.

### Defining sustainability innovations

When seeking to comprehend sustainability innovations, transition and innovation studies offer a conceptual understanding of *innovative* aspects such as innovation types and development processes, whereas the sustainability assessment literature elaborates on what distinguishes *sustainability* innovations from what we may call *conventional* examples, i.e., innovations that have no intention and no concrete impact in terms of sustainability. Most innovation studies build on Schumpeter’s (1934) seminal conceptualisation, which demonstrated that innovations, in opposition to inventions, refer to novelties applied and diffused, including product, process and organizational novelties, both radical and incremental. Business-driven understandings of innovation focus on product, process, organizational and marketing—or, in a new categorisation on product and business process innovations (OECD/Eurostat 2018). In addition, recent innovation studies have shed light on a more capacious understanding of innovations, including social, behavioral, technological or rural innovations (Gamito and Madureira 2019). In a comprehensive understanding, innovations respond to specific social needs and desires, or even (re-)define needs and desires (Baker and Mehmood 2015). Fagerberg (2009) underlined the systemic aspects of innovation, which are embedded in and shaped by the specific environment or context in which they develop. In these complex systems, innovations develop as multi-scalar processes that unfold across various socio-technical (sub-)systems (Geels 2019), and in what follows they encompass collective processes of inventing, experimenting, developing, supporting and spreading, all of which therefore involve a multiplicity of actors.

Sustainability innovations have the same characteristics as those described above, but, as the name suggests, they particularly encapsulate sustainability outcomes. The guiding assumption is that sustainability is a comprehensive and holistic concept used to describe the preservation and strengthening of social-ecological systems in the long term, via multiple reinforcing steps (Gibson 2006). More specifically, sustainability refers to social–ecological systems capacity to maintain and restore human wellbeing, social equity and environmental integrity (Leach et al. 2010). Against this backdrop, sustainability innovations do not simply mitigate negative impacts, but rather should induce net positive outcomes. Still, sustainability also concerns processes and means rather than only targets and results (Meadows 1998), in which case precaution and justice principles must be addressed. Henceforth, sustainability remains a normative concept, for which concrete understandings and applications are context-dependent and shaped by place-specific environmental, political, social and cultural conditions (Gibson 2006; Leach et al. 2010). Sustainability innovations therefore produce positive outcomes in terms of social–ecological integrity and equity in specific contexts.

In this study, we propose the following definition: sustainability innovations are multi-scalar, multi-actor processes that develop new ways to define and meet social needs and induce positive outcomes in terms of social–ecological integrity and equity—in a specific and complex social–ecological context. This definition casts light on four prominent aspects that constitute the basis of our analytical framework: actors, processes, outcomes and context.

### Developing a sustainability innovation framework

The central objective of this study is the conceptualisation of an analytical framework for sustainability innovation, which we call sustainability innovation framework in the following. This framework aims to provide a comprehensive and generic set of relevant variables that potentially induce influence on innovation development and outcomes, to enable the analysis and cross-case comparison of focal, individual sustainability innovations, in a context-sensitive manner. The sustainability innovation framework thereby conceptualizes an innovation systems by distinguishing various system dimensions and potential influencing variables. Relevant dimensions and variables, as well as their interactions, were identified deductively with help of a literature research. Conceptually, we built on prominent systemic approaches in the fields of social–ecological systems (McGinnis and Ostrom 2014; Ostrom 2009), sustainability assessments (Gibson 2006; Luederitz et al. 2017) and innovation and transitions studies, including the MLP (Geels 2002, 2011, 2019), transition management (Frantzeskaki et al. 2012; Loorbach 2007; Wittmayer et al. 2018), TIS (Cooke 2010; ﻿Bergek and Mehmood 2015; Hekkert et al. 2007) and GeoST (Binz et al. 2020; Coenen and Morgan 2020). This literature research resulted in the identification of four system dimensions (actors, process, outcomes and context) and 31 variables that potentially influence sustainability innovation development. Related concepts were synthesized to reach a comprehensive and yet clear set of variables. The resulting framework was evaluated, applied to two case studies and adjusted in an iterative manner over a year. The following sections display the concepts and literature bodies on which the framework specifically draws.

#### Four dimensions for analysis: context, actors, process and outcomes

The sustainability innovation framework comprises an initial analytical tier of four prominent dimensions, namely context, actors, process and outcomes, which are inspired by the social–ecological systems (SES) framework (McGinnis and Ostrom 2014; Ostrom 2007﻿, 2009). The SES framework provides analytical guidance in assessing variables that influence the performance of institutional arrangements for the use of natural common goods in focal social–ecological systems. Furthermore, it details resource systems, resource units, governance systems, actors, interactions and outcomes, while related ecosystems, as well as social, economic and political settings, are considered as an influencing contexts for specific social–ecological systems (McGinnis and Ostrom 2014).

In accordance with the SES framework, albeit with a particular focus on collective processes of innovation rather than natural resource use, the sustainability innovation framework thus acknowledges the central role of *actors* in the development of sustainability innovations*.* Actors in this context refer to stakeholders and organizations who invent, cooperate, support, use, adopt or even hinder a focal innovation *process.* In line with the SES framework, *processes* are conceptualized as actions and interactions within the actor group and with other system dimensions. Following these interactions, *outcomes* are the results of innovation processes in a given *context*. In contrast with the SES approach, which focuses on biophysical and governance systems (Ostrom 2009), we adopt herein a more generic approach to the settings in which innovative actors interact and experiment. As proposed by the innovation systems approach (Markard et al. 2015) and GeoST (Binz and Truffer 2017), innovations are shaped by their local and global contexts. Different aspects will appear relevant in different cases, so we refer to general settings as *context* and include a large range of potentially relevant aspects, such as political, social, technological, economic and biophysical settings, as detailed in the next section. Note that, in line with the SES framework, the sustainability innovation framework seeks to guide case analysts in unraveling relevant factors and characteristics in given situations (Ostrom 2007). We acknowledge systemic multi-level influences—but focus on context-specific individual innovations rather than broader regime or system change processes (Geels 2019).

#### Context

The context dimension of the analytical framework refers to all external influences and conditions that shape, support or hinder sustainability innovations. Specific contextual conditions may constitute direct barriers or opportunities for innovation, such as regional lock-ins, due to existing industrial infrastructure (Geels and Schot 2007), or new policy programs and funding opportunities (Shove and Walker 2007). The context also sets a baseline against which processes may be considered novel and sustainable (Gibson 2006). For instance, while a product or behavior can be innovative in a given place at a certain time, the same product or behavior might as well go unseen in another context (Fagerberg 2009). Similarly, some form of novelty may bring about sustainability improvements in a given context, while it may be a setback in another. Various systems analysis approaches focus on particular context conditions. The SES framework, for instance, emphasizes biophysical conditions (McGinnis and Ostrom 2014), while the MLP focuses on socio-technological system conditions (Geels 2011). TIS recognizes the influence of governance and institutions on the development of technologies, albeit it falls short of biophysical, cultural and social aspects (Asheim and Coenen 2005). GeoST calls for a more comprehensive understanding of the place-specific conditions of innovations (Coenen et al. 2012). This approach addresses space and place in a relational manner, including histories, cultures, actors, materials and their interactions (Binz et al. 2020). The sustainability innovation framework thereby acknowledges that the framing of relevant scales, influencing variables and the bounding of focal sustainability innovations in their relevant context, as part of complex social-ecological systems, may vary depending on research objectives, personal interpretations and normative assumptions (Leach et al. 2010). To encompass the variety of potential influences on sustainability innovation development, we propose a set of generic context variables, which might play out very differently across cases and need to be defined and carefully reflected upon while framing the case analysis: ecological (C1), political (C2), economic and financial (C3), social and cultural (C4), technological and infrastructural (C5) and other contextual variables (C6).

#### Actors

The actor dimension of the sustainability innovation framework encompasses all relevant stakeholders and organizations that innovate, support, cooperate in, use, adopt or even hinder a specific sustainability innovation. The social-ecological systems framework provides a starting point for detailing this dimension, by highlighting the influence of the number of actors, their characteristics, their norms, values and trust in terms of collective decision-making (McGinnis and Ostrom 2014). Furthermore, looking at innovations rather than at the use of natural common goods, the innovation systems approach acknowledges the prominent role of organizations, such as firms, research and administration, and insists on distinguishing actors types, e.g., private, public and individuals (Asheim and Coenen 2005). As proposed by Westley et al. (2013) and Asheim and Coenen (2005), actors’ resources, knowledge and roles, including inventors, supporters and advocates, have been identified as potentially relevant variables for case study analyses. In what follows, the actor dimension features seven variables that guide the analysis of involved people and organizations by type (A1), role (A2), resources and competences (A3), interests and values (A4), trust and commitment (A5), number (A6) and other characteristics (A7).

#### Process

The process dimension of the sustainability innovation framework refers to what actors do and how they do it. Within the field of sustainability transitions, the transition management approach has thoroughly explored change processes by accompanying transition processes in the making of and learning from practice, for instance in cities (Frantzeskaki et al. 2020) or in the energy sector (Kemp et al. 2007). Wittmayer et al. (2018) distinguish the major phases and activities of transition processes, including building teams and partnerships, problem analysis and framing, envisioning and setting goals, acting and experimenting and reflecting. Furthermore, the social-ecological framework highlights the shaping effects of conflicts in decision-making, such as in natural resource use and conflicting interests among stakeholders (McGinnis and Ostrom 2014). The technical innovation systems approach further underlines the need for lobbying and gravitating toward other organisations’ higher governance levels, as in the case of novel technologies, such as biofuels or renewable energies, which require regulatory adjustments and social and political support (Bergek et al. 2015). Consequently, we synthesize these learnings and sustainability innovation processes into eight variables: envisioning and goal-setting (P1), action and experiments (P2), learning and sharing knowledge (P3), decision-making (P4), building networks and partnerships (P5), conflicts (P6), advocating, marketing and lobbying (P7) and other potential processes (P8).

#### Outcomes

The fourth dimension of the sustainability innovation framework refers to outcomes, which encompasses innovation types, as well as its development stages and sustainability-related results. Outcomes include the type of novelty at stake, for example social, institutional, behavioral and organizational innovations (Gamito and Madureira 2019). In this dimension, we also refer to development stages such as amplification and spreading processes and results (Lam et al. 2020). Besides innovation type and the development stages, the outcome dimension is much less explored in innovations and transition studies. Whereas some authors highlight processual aspects, such as responsibility, participation, learning and systemic and long-term thinking (Schlaile et al. 2017; Wittmayer et al. 2018), no comprehensive analytical set is provided by any of the analysis frameworks at hand.

The sustainability innovation framework thus builds on learnings from the sustainability assessment literature to expand on outcomes. The guiding assumption is that sustainability is a comprehensive, holistic and normative concept employed to describe the protection and strengthening of social–ecological systems in the long term. Social–ecological integrity requires multiple reinforcing steps, which maintain conditions for human wellbeing as being interdependent with biophysical systems and life support functions (Gibson 2006). We now follow the proposed list of sustainability features provided by Gibson (2006) and Luederitz et al. (2017), and we use them as potential outcomes of sustainability innovations. Innovations may cause structural changes in physical and social structures (Luederitz et al. 2017), while resource maintenance and efficient use ensure the long-term viability of social–ecological systems (Gibson 2006). In addition, livelihood sufficiency and opportunity allow individuals and communities to strengthen their quality of life and wellbeing (Luederitz et al. 2017), and intra- and intergenerational equity guarantee equal opportunities and decent life conditions (Luederitz et al. 2017). Social–ecological stewardship and democratic governance strengthen individuals and communities in their commitment and ability to promote sustainability and democratic decision-making (Avelino et al. 2020; Luederitz et al. 2017). Precaution and adaptation account for risks and unforeseen changes, and they improve the ability to prevent or react to them (Gibson 2006). As a result, the outcomes dimension features ten variables: innovation type (O1), amplifying and spreading (O2), structural changes (O3), social–ecological integrity (O4), livelihood sufficiency and opportunity (O5), intra- and intergenerational equity (O6), resource maintenance and efficiency (O7), social–ecological stewardship and democratic governance (O8), precaution and adaptation (O9) and other potential outcomes (O10).

**Fig. 1:**
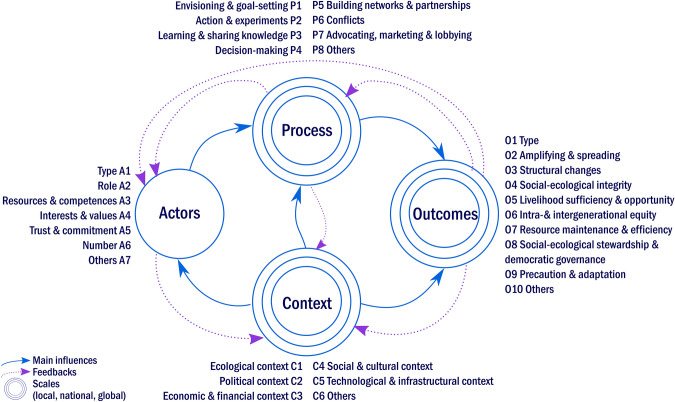
Sustainability innovation framework. Dimensions, variables and interactions

In summary, the sustainability innovation framework consists of four system dimensions—context, actors, outcomes and processes—and a set of 31 potentially influencing variables related to these dimensions (Fig. 1). The framework depicts major influences and interactions, which unfold across these system dimensions and scales (Binz and Truffer 2017; Coenen et al. 2012; Hielscher et al. 2022). Acknowledging the multi-scalar character of sustainability innovation, the analytical framework particularly considers actors’ interactions, spanning from local to global scales and contexts, with a focal starting point on individual sustainability innovations (Köhler et al. 2021).

## Methods

To test the applicability and accuracy of the sustainability innovation framework for analytical purposes, two sustainability innovations were selected as case studies in Schorfheide-Chorin Biosphere Reserve (Germany), a peri-urban to rural region located to the northeast of Berlin. As biosphere reserves, nominated by UNESCO, aim to function as model regions for sustainable development, they provide compelling study sites for sustainability innovation. Our cases were the Solar Explorer, a solar-powered catamaran developed for research and education purposes, and Brodowin Ecovillage, a large-scale, organic farm stemming from a former collectivist land cooperative.

### Case selection

The two cases were selected during a regional inventory of sustainability innovations at the Schorfheide-Chorin Biosphere Reserve. In the first phase of the inventory, five semi-structured expert interviews were conducted with regional informants about sustainability innovations and prominent projects in the region. Interviewees were representatives of local administrations, the biosphere reserve, a local association and a university (Table 1). From the inventory, two cases were selected. A first criterion was to select well-known, real-world, stabilized innovations in the region, which were mentioned by several of our regional informants. A second criterion was to select cases that differed in terms of goals, sectors and innovation type, thus allowing the framework to be applied to varying contexts for cross-case comparison. The first case is the Solar Explorer, a sun-powered catamaran developed in 2011 for educational purposes by a wide range of public and private actors. The second case is Brodowin Ecovillage, a social innovation, which started with the creation of a large-scale biodynamic farm after German reunification and later developed through multiple incremental changes. Both initiatives were pioneers at a time when solar power and biodynamic farming were very much in their infancy.

**Table 1:** List of interviews

ID	Organization	Function	Topic	Length	Date
SCi1	Schorfheide-Chorin Biosphere Reserve	Employee	Regional SI inventory	01:00	03/03/2021
SCi2	Schorfheide-Chorin Biosphere Reserve	Employee	Regional SI inventory	01:00	15/03/2021
SCi3	University for Sustainable Development Eberswalde	Scientific staff member	Regional SI inventory	00:20	23/03/2021
SCi4	Regional Planning Department	Employee	Regional SI inventory	00:40	24/03/2021
SCi5	Schorfheide-Chorin Biosphere Reserve	Employee	Regional SI inventory and Solar Explorer	01:10	09/04/2021
SCÖB1	Ökodorf Brodowin GmbH	Employee	Brodowin Ecovillage	01:10	20/04/2021
SCSE1	Kulturlandschaft Uckermark e.V	Executive director	Solar Explorer	01:10	20/04/2021
SCÖB2	Ökodorf Brodowin e.V	Board member	Brodowin Ecovillage	01:40	04/05/2021
SCSE2	Kulturlandschaft Uckermark e.V	Former employee	Solar Explorer	00:50	05/05/2021

### Qualitative expert interviews and data analysis

Qualitative data were collected on each sustainability innovation through online semi-structured expert interviews. Following a request for an interview sent to three prominent actors per case, five agreed while one person involved in the Brodowin Ecovillage declined. Contacted stakeholders were employees, a board member of a local association and a representative of the biosphere reserve, each of whom had in-depth knowledge of their respective cases. The interviewees had different functions and positions, and each was employed by a different organization (private firm, NGO, public administration), which ensured a variety of opinions and perspectives on each case. The analytical framework was used to structure the interview guide along the four sustainability innovation dimensions: context, actors, process and outcomes. Variables were transposed into questions along the four mentioned dimensions. Additionally, questions were included about the perceived influence of the vicinity of the capital city and of the biosphere reserve, to elaborate on potential specific influences. Interviews lasted about one hour on average. The audio recordings were transcribed and then coded with MaxQDA for qualitative content analysis (Mayring 2007), using the sustainability innovation framework dimension and variables for categorisation. Consequently, we identified relevant variables and drew a comparison of influences and characteristics across the two cases.

## Results

### The Solar Explorer: a technological and service innovation

The Solar Explorer is an 18-m-long research and educational solar-powered catamaran, which has been based on the Werbellinsee in Schorfheide-Chorin Biosphere Reserve since 2011. In 2006, the original idea was developed by a network of diverse actors: a boat constructor, the biosphere reserve administration, a local association, the German society for solar energy, a nearby university and other supporters had the boat built in 2011. Since then, the Solar Explorer has been dedicated primarily to education for sustainability, and equipped with high-tech research equipment for ecological research, thereby becoming a well-established technological and product innovation. What factors have influenced the development and the outcomes of the Solar Explorer? What are its outcomes? The analysis of the interview material casts light on the following variables, as described in the following paragraphs.

#### Context

The development of the Solar Explorer happened in a context of growing awareness (C4) of global changes (C1), at a time when solar technology was on a very small scale globally (C5). Locally, the biosphere reserve and the clear-water lake ecosystem (C1) inspired the actors to commit to sustainability. The attractive location for tourism and outdoor recreation, aligned with the will of schools to support education for sustainability (C4), represented a good opportunity to reach out to visitors. Funding opportunities by public (C2) and private donors at regional and national levels (C3) enabled the project’s implementation. However, the regional mobility infrastructure (C5) prevented access to the Solar Explorer by public transportation—and thus participation by some schools. Furthermore, the Covid-19 pandemic (C4) put a stop to all educational programs in 2020.

#### Actors

A few regional visionaries and leaders developed the project by reaching out to resource and knowledge brokers and to implementers (A2). Their diverse interests (e.g., solar technology, education for sustainability, rural development) (A4) gave way to the multiple facets of the sustainability innovation. The availability of professional competences (e.g., solar technology, education) and soft skills (e.g., networking, facilitating) was considered a crucial resource (A3). Understanding of local structures and mentalities was mentioned also as a requirement for successful implementation (A3). Funding was constantly searched for and made the innovation and its stabilization possible (A3). Strong commitment by many actors (A5) prevented failure, due, for instance, to a lack of funding.

#### Process

Throughout the whole process, a crucial activity involved advocating for the project to find support for funding at the local and national levels, to inform schools about the program and to search for potential partners for the development of new projects (P7). Additionally, actors focused on implementation (i.e., boat construction and maintenance, educational program) (P2). Nevertheless, the continuous search for funding required a great deal of energy and therefore strongly limited monitoring (P3) and the development of new ideas (P1). The Solar Explorer was thereby ever more focused on education for sustainability rather than on ecological research, and the research equipment has been less used than envisioned.

#### Outcomes

Against this backdrop, the Solar Explorer became a technological product innovation at a global level and a service innovation for education for sustainability in the region (O1). This resulted in sustainability outcomes, as it fostered local resource maintenance and efficient use through solar technology for mobility (O7), thereby becoming a flagship project for the biosphere reserve administration and an inspiring demonstration project for solar technology. The Solar Explorer also participated in ecological stewardship (O8) through showcasing solar technology and through awareness-raising and knowledge transfer at the local to global levels, with a strong focus on local school children. The project also fostered actor empowerment and capacity-building. Indeed, the Solar Explorer gave way to further experimental projects in the field of regional sustainable mobility, with the commitment of many of the involved actors (O2). For instance, following projects in the biosphere reserve targeted e-bike mobility and public transportation networks. Finally, the Solar Explorer participated in strengthening regional intergenerational equity by promoting high-quality educational programs in a low-density rural region in which such offerings are rare (O6). Indeed, several of our interviewees underlined the difficulty for such a rural area to provide out-of-school educational activities in comparison to well-connected and well-financed schools in urban areas.

### Brodowin Ecovillage: a social, multi-faceted innovation

The Brodowin Ecovillage is a regionally well-known sustainability innovation in the agricultural sector. It is a private large-scale biodynamic farm developed in the early 1990s on former collectivist cooperative land (in German *Landwirtschaftliche Produktionsgenossenschaft*). After the regime change, agricultural land around the village could have been redistributed to its former owners, but about 80 local farmers took the decision to continue with a collective scheme via a private company, with the goal to preserve jobs and to transition to biodynamic farming. The name “Ecovillage” was adopted, as almost all former landowners and villagers committed to the decision. This initial social innovation gave way to multiple other novelties. Most importantly, the Brodowin Ecovillage implemented nature protection measures. Further incremental innovations in processing, commercialisation and low-carbon delivery were also developed, and so the scheme was thus considered an organizational and a social innovation, which grew as a result of further product, marketing and process innovations. What factors influenced the emergence, the development and the outcomes of Brodowin Ecovillage?

#### Context

The original catalyst for the creation of the Ecovillage was the national political transition in 1989 and the early 90s (C2). The transition raised local questions relating to the reprivatisation of land. Although sustainability and organic agriculture were niche concepts at that time at a global level (C4), the simultaneous creation of the biosphere reserve (C2) inspired involved actors to opt for organic farming. The growing demand for and acceptance of organic agriculture (C4) in the ensuing decades supported the stabilization of the farm. Furthermore, its proximity to Berlin offered regional market opportunities (C3), while the low-density rural region (C4) has allowed surface expansion in later stages. Very recently, the COVID-19 pandemic (C4) saw an increase in demand for organic products and therefore economically benefitted the Ecovillage.

#### Actors

The original discussion about a potential redistribution of agricultural land involved former employees of the collectivist cooperatives and other village actors. After the creation of the private farm to oversee the project, other actors committed to the idea, such as the local Ecovillage association (in German Ökodorf Brodowin e.V.), regional and national private partners (e.g., neighboring farms and donors) and the biosphere reserve administration (A1). This actor constellation crystallized around multiple interests (A4), such as ensuring local jobs and economic viability, promoting sustainable land use and nature protection and enhancing quality of life. Actors played different roles in terms of leadership, facilitating, implementing and resource- and knowledge-brokering (A2). The availability of funding (A3), provided by the directors, enabled the stability and development of the farm throughout difficult times. The interview partners considered trust, strong commitment (A5) and competent personnel (A3) as critical success factors. Professional expertise, not only in agriculture, but also in ecology and nature conservation, as well as soft skills like facilitation and networking (A3) also enabled the success of the project.

#### Process

The interviewees shed light on challenges and crises as catalysts for innovation throughout the whole process, e.g., political transition. Hence, a pattern of envisioning solutions and setting goals (P1), doing experiments (P2) and learning from them (P3) was repeatedly reported by our interviewees. An example was the development of processed products, which first started as a limited activity but soon expanded to make use of leftovers and surpluses, which then led to more recipes and more products. Experiments were made possible by open decision-making processes (P4), such as the initial decision to work collectively or the common yearly design of nature protection measures together with the local association.

#### Outcomes

Over the last few decades, the variety of activities has ensured economic stability (O9) and led to multiple outcomes, including the original social innovation and follow-up product and process innovations (O1). Locally, the farm grew in area and net benefits and extended its panel of products to processed food, dairy and meat, vegetables and fruits in weekly subscription. Incremental innovations were made in products and processing (O2), and structural changes were made to the local landscape, which turned from conventional large-scale agriculture to organic farming with protected patches of land for biodiversity (O3). In turn, this enhanced social–ecological integrity through the preservation of habitats for fauna and flora while creating attractive landscapes for locals and visitors—and thus enhancing human quality of life (O4). Resource maintenance and efficient use were strengthened through the processing of leftovers from the vegetable, fruit and meat production, the use of solar energy on site and CO_2_ emission reductions in delivery through partnerships with bike delivery services in Berlin (O7). The high employment rate on the organic farm fostered local livelihood sufficiency (O5), albeit the spread and growth of the farm also fostered land concentration and the dependency of smaller farms on their large-scale neighbor, for instance for dairy processing. To some, the Ecovillage came to resemble an agricultural monopoly. Nonetheless, as it attracted tourists, new business opportunities were created for local housing and gastronomy entrepreneurs (O5). Yet increasing tourist flows from Berlin toward what became an eco-tourism attraction—coupled with underdeveloped public transportation infrastructure—increased pressure on the local population and environment, due to high numbers of private cars in the village and surrounding areas. Finally, social–ecological stewardship (O8) was strengthened through knowledge production and capacity-building within the actor constellation, leading to amplification and spreading at the regional and national levels (O2). Indeed, the implementation of nature protection measures was institutionalized on the farm and later applied in follow-up agro-ecology projects in the region and beyond, led partly by the same actors (O2).

### Cross-case comparison

Through multiple actors and processes, and with various sustainability outcomes, both cases epitomized the proposed definition of sustainability innovations. Nonetheless, the cases displayed clear differences in the variables and conditions that influence the emergence, development and outcomes of each sustainability innovation.

**Fig. 2:**
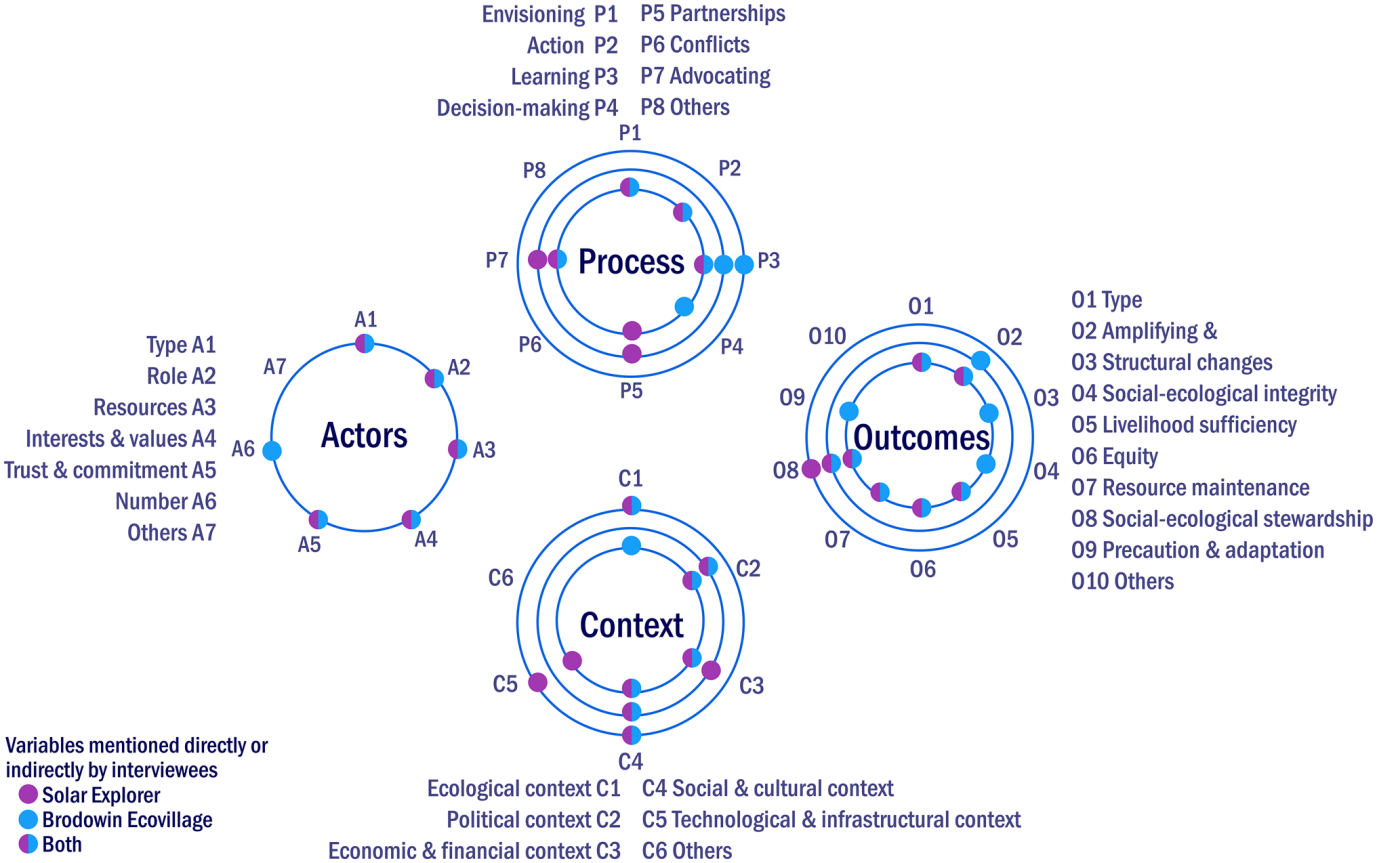
Solar Explorer and Brodowin Ecovillage: relevant analytical variables

As for commonalities, both cases emphasized the importance of a trusting, committed actor network that would support the sustainability innovation through complementary roles and competences, as well as with secure funding (A1, A2, A3, A4 and A5). Actor diversity fostered diverse goal-setting, including social, ecological and economic aspects. As for processes, envisioning, experimenting, learning, advocating and building partnerships were most influential in both cases (P1, P2, P3, P4 and P7). Nonetheless, little time could be dedicated to learning and envisioning by the Solar Explorer team, which was considered a limiting factor for development. Both innovations benefited from windows of opportunity to develop; for example, changes in land tenure systems enabled the creation of the Ecovillage. Both innovations co-evolved with their contexts (C1, C2, C3 and C4), such as organic farming with a nearby urban market. With regards to outcomes, the cases displayed different results, but both produced a panel of multiple outcomes. For example, the Solar Explorer aimed to strengthen not only social–ecological stewardship, but also livelihood opportunities and equity. Figure 2 highlights the common set of influencing factors, in particular in the actor and process dimensions, which were mentioned by interviewees in each case. The outcome dimension showed more diversity between the two cases.

Besides commonalities, it was also possible to detect differences between the two cases by applying the analytical framework. Overall, the Solar Explorer and Brodowin Ecovillage differed in terms of secure funding, and consequently in their capacity to achieve certain goals, and therefore in their outcomes. As for the Ecovillage, the commitment of diverse actors fostered the integration of economic viability with sustainable measures and local livelihood opportunities. These diverse goals resulted in various outcomes, including structural changes, improvements to social–ecological integrity, job preservation and precautionary measures. Secure funding thereby proved crucial. Conversely, the Solar Explorer project lacked secure and permanent funding, and so the team focused on building partnerships and advocating. This, in turn, limited time for learning and envisioning, finally resulting in less diverse projects and outcomes. As a result, the Solar Explorer resulted most prominently in awareness-raising and social–ecological stewardship.

## Discussion

In this article, we set out to provide a specific and wide-ranging characterization of sustainability innovation and to develop an analytical framework, which we tested in two case studies. In the following, we discuss the framework’s analytical capacity and insights gained from its application on two case studies, our contributions to transitions and innovations studies and the methodological limitations of this study.

### Specific characteristics of sustainability innovations

The case studies demonstrated that the proposed analytical framework is applicable, generic and comprehensive and that it applies well to multiple types of sustainability innovations at stake. The framework enabled us to study the influencing factors, characteristics and outcomes of two cases of sustainability innovations in a comparative manner, thereby allowing us to interpret the cases material in terms of commonalities and differences.

The cross-case comparison provided empirical confirmation of a key set of actor- and process-related conditions, which turned out to be crucial for innovation development. In accordance with transitions and innovations studies, resource availability (Hekkert et al. 2007), various competences and roles (Wittmayer et al. 2017), trust and commitment, building partnerships and advocating (Wittmayer et al. 2018), envisioning, experimenting and learning (Asheim and Coenen 2005) all proved crucial in fostering or—when missing—limiting our focal innovations. Moreover, actors’ capacity to understand and navigate their contexts, i.e., to use opportunities, co-create enabling conditions and overcome barriers, appeared to have a great impact on success and sustainability outcomes. For example, the close vicinity of Berlin, the capital city, created opportunities and challenges alike for both the Solar Explorer and the Ecovillage. Further studies are needed to understand sustainability innovation dynamics in differently urbanized areas, for instance in peri-urban biosphere reserves (Harris et al. 2019). Our cases also suggested a strong influence of the biosphere reserve on innovative activities; yet, as shown by Kratzer (2018), we expect that our cases were exceptional in that regard and that most regional innovations are less impacted by the local biosphere reserve.

Furthermore, both cases illustrated that sustainability innovations develop as bundles of interdependent, entangled novelties (Avelino et al. 2019). For example, the Ecovillage started with social and organizational innovation, followed by various, successive product, process and market novelties to adapt to evolving markets and opportunities. These innovation entanglements relate to cluster effects, although the latter are more often reported at the regional level and across many organizations (Asheim and Coenen 2005; Fagerberg 2009). Built capacities and learnings often open up the way for further innovations at the regional or organizational level (Luederitz et al. 2017). More specifically, though, sustainability innovations, being disruptive in current deteriorating social–ecological systems, require changes in their own context, for instance in values, habits and institutions (Engels et al. 2019). Consequently, it is not surprising that they encapsulate multiple novelties, or innovation types, to fully unfold (Wittmayer et al. 2020, 2022). With regard to the specificity of sustainability innovations in contrast to other innovation types, this study therefore showed that it resides in outcomes rather than in another particular type of novelty. For example, the Solar Explorer comprised technological and organizational novelties, but it was most specific in dedicating these novelties to social–ecological stewardship and educational programs for local, rural schoolchildren. In that sense, sustainability innovations differ from product, process or organizational innovations, which are substantially characterized by their novelty type, rather than by outcomes.

Prospectively, our study pledged for additional studies of what we may call “discreet” innovations. These are less eye-catching—but no less insightful—innovations than, say, socio-technical changes in mobility or energy systems or other prominent global technological innovations (Avelino et al. 2019; Feola 2020; Nicolosi et al. 2018; Wittmayer et al. 2022; Köhler et al. 2019). Sustainability innovations can be part of a trend of similar projects rather than global pioneers (Nicolosi et al. 2018). For instance, the Ecovillage, with its large-scale organic farming scheme, was not unique but nevertheless proved innovative in its regional and even national context through the creation of new social, cooperative arrangements and through a combination of employment, biodiversity conservation and organic farming. Many such discreet innovations ought to exert adaptive capacities and be innovative in their local contexts (Fagerberg 2009). The participation of such discreet, local innovation to niche development (Geels 2011) remains to be explored and promises compelling insights into space-sensitivity and context-dependency of societal, systemic change processes (Gamito and Madureira 2019).

### A generic, systems-based framework

The analytical framework was built on conceptual insights from transition studies, the SES framework and sustainability assessments. Using these frameworks as the conceptual foundation for analysis proved insightful, as they cover important dimensions, such as governance systems, biophysical, socio-technical systems, and address different levels of analysis, from small-scale, individual social–ecological arrangements or innovations to broader scale, systemic change processes.

The study demonstrated that the sustainability innovation framework is generic and covers a comprehensive range of variables. In comparison, other frameworks tend to address particular dimensions. The SES framework focuses on social–ecological systems, thereby highlighting governance systems (McGinnis and Ostrom 2014). The MLP and TIS target technological innovations and therefore concentrate on socio-technical arrangements across broader, multi-scalar change processes (Bergek et al. 2015; Geels 2011). In contrast, the proposed sustainability innovation framework included a large number of fined-grained variables, potentially relevant for focal, individual sustainability innovations. Such a generic and fined-grained approach proved useful, because sustainability innovations, as demonstrated above, may encompass various types of small-scale innovations, including technological, behavioral, process novelties, etc﻿ (Hielscher et al. 2022). Moreover, as sustainability innovations are normatively loaded, their understanding may vary across contexts, which therefore requires a broad analytical approach with regard to outcomes (Ramos-Mejía et al. 2018). In this exploratory study, we thus tested a large range of variables, to better understand the multi-facetted concept of sustainability innovation.

Furthermore, the framework expounded on the sustainability outcomes dimension, which has received little attention so far in the transitions and innovation literature (Feola 2020; Truffer et al. 2022). For instance, the Ecovillage resulted in various beneficial outcomes, from structural landscape changes to livelihood sufficiency, although some contentious monopolistic structures were criticized as well. The critical evaluation of some interviewees underlined that understandings of sustainability—and consequently sustainability innovations—may vary subjectively and across contexts. This underpins the need for thorough analyses of innovations’ outcomes. In this regard, further elaboration should take into consideration approaches other than the ample research—and underlying understandings of sustainability innovations—from the Global North, which we have largely used in our research (Nesari et al. 2022; Preuß et al. 2021; Ramos-Mejía et al. 2018).

### Methodological limitations

Our study faced methodological limitations typical of social empirical qualitative research. In general, it posited that our interviewees’ perceptions of past and current events would provide comprehensive information; however, they did not mention all of the potential variables. Although it is possible that some variables indeed had no relevance, a lack of information could also mean that characteristics or influences were not perceived or reported by our interviewees, or that the variables had been overseen during the analysis. For instance, influential factors might have been omitted or under-evaluated, e.g., if they were taken for granted or related to long-past events. Although interviewees were carefully chosen, so as to provide in-depth knowledge through a diversity of perspectives on each case, their positions as employees or active supporters in the cases might only cast a certain—positive—light on the projects.

Sustainability, similar to the concept of innovation, is a normative, value-driven concept and can be interpreted in various ways across different contexts (Meadows 1998; Schlaile et al. 2017). In our empirical study, we thus built on our interviewees’ knowledge and judgment to identify, first, local sustainability innovations and, second, their positive—or negative—outcomes. Note that sustainability innovations may have destructive consequences, even though they challenge unjust and destructive systems (Avelino 2021; Fougère and Meriläinen 2021). For example, the large-scale concentration of agricultural land has arguably reinforced monopolistic land ownership and thus affected intra-generational equity.

While the case study material provided ample information about the relative importance of specific factors, the framework in itself does not yet provide an explanatory pattern in sustainability innovation development and outcomes. Rather, the framework provides a comprehensive set of potentially relevant variables as guidelines for the case analysis and interpretation. It was therefore only possible to assess the relative influence of the mentioned variables through interview material analysis, although a detailed weighting scheme applied during a discourse analysis on larger n data could provide insights about the most influential variables in a cross-case comparison scheme. Thereafter, we could neither provide insights about the mechanisms through which the mentioned variables exerted influence over focal innovations nor assess in how far perceived influence may relate to causal relationships. As argued by Coenen et al. (2012), historic or geographic interpretations of innovation journeys do not unravel causality links. More generally, the framework does not provide a blueprint for identifying what necessarily supports or hinders sustainability innovation.

## Conclusion

The analytical framework synthesized influencing variables and the characteristics of single sustainability innovations into four analytical dimensions: context, actors, process and outcomes. The specificity of sustainability innovations resides in their positive outcomes, in terms of social-ecological integrity and equity, so the outcomes dimension therefore detailed relevant variables for analysis. Furthermore, our empirical cases demonstrated that the framework is comprehensive, generic and applicable to a wide range of novelty types, rather than merely technological innovations. As a result of our case studies, we were able to identify preliminary patterns and a set of key common factors. Nonetheless, further empirical research is required on various sustainability innovations and in different settings, to identify innovation patterns across contexts. In this regard, the sustainability innovation framework provides the opportunity to develop a typology and build up a comparative scheme about sustainability innovation archetypes, development patterns, contextual influences and potential barriers. Such learnings will in turn inform innovation governance, by underpinning the design of enabling contexts. The need thus remains to thoroughly examine the outcomes of innovations at stake, and to go beyond mere declarations of intentions, to support truly transformative sustainability innovations.

## Data Availability

Not applicable.
